# Analysis of environmental risk factors, prevalence, and associated symptoms of *Giardia intestinalis* infection across age groups in Poland: a survey-based study

**DOI:** 10.1007/s00436-026-08631-3

**Published:** 2026-02-18

**Authors:** Sylwia Klimczak, Kacper Packi, Alicja Rudek, Marcin Kurowski, Agnieszka Śliwińska

**Affiliations:** 1 Department of Nucleic Acid Biochemistry, Medical University of Lodz, Lodz, 92-213 Poland; 2 AllerGen Center of Personalized Medicine, Piotrkow Trybunalski, 97-300 Poland; 3 Department of Immunology and Allergy, Medical University of Lodz, Lodz, 92-213 Poland

**Keywords:** *Giardiaintestinalis*, Intestinal protozoa, Parasitic infection, Epidemiology, Poland, Population-based survey

## Abstract

*Giardia intestinalis* is one of the most common intestinal protozoa worldwide, yet its true occurrence is challenging to estimate due to frequent asymptomatic or nonspecific presentations and limited diagnostic access. Epidemiological data from high-income Central European countries remain scarce. This study aimed to assess the proportion of *G. intestinalis* infections and to identify environmental, behavioral, anthropometric, and clinical correlates across age groups in Poland. A cross-sectional, survey-based study was conducted among 518 individuals presenting for parasitological stool examination at a certified diagnostic laboratory. Participants provided stool samples and completed standardized epidemiological and clinical questionnaires. Associations between exposures, anthropometric variables, and symptoms and infection status were analyzed using χ² tests and logistic regression, stratified by age (children, adolescents, younger adults, older adults). *G. intestinalis* infection was identified in 24.7% of participants in this clinically referred, self-selected cohort. Due to the sampling method, this value cannot be generalized to the general population. In the total cohort, self-reported bloating, supplement intake, and having children in preschool or primary school were associated with lower infection odds, whereas constipation increased risk; however, these associations likely reflect cohort-specific behavioral and clinical characteristics rather than causal effects. Age-specific patterns were observed: in younger adults (18–40 years), previous giardiasis and pet ownership were significant risk factors, while dietary changes were protective. In adults > 40 years, infection correlated with anthropometric measures and supplement intake, the latter showing an opposite direction of association relative to the overall cohort. Symptom profiles varied by age, with gastroesophageal complaints in children, oligosymptomatic presentation in younger adults, and constipation with systemic symptoms in older adults. A high proportion of *G. intestinalis* infections was observed among individuals undergoing parasitological diagnostics in Poland. These findings reflect patterns within a clinically referred population and do not provide estimates of national prevalence. The observed age-dependent symptom profiles and associations with behavioral and host-related factors highlight the need for improved diagnostic vigilance in clinical settings. Population-based studies using representative sampling and standardized diagnostics are required to determine prevalence in the general population and clarify causal pathways.

**Supplementary Information:**

The online version contains supplementary material available at 10.1007/s00436-026-08631-3.

## Introduction

*Giardia intestinalis* (syn. *G. lamblia*, *G. duodenalis*) is an intestinal protozoan widely distributed worldwide, responsible for giardiasis—one of the most common parasitic diseases of the gastrointestinal tract in both humans and animals (Adam 2021). Transmission occurs primarily via the fecal–oral route through the ingestion of water or food contaminated with cysts, as well as through direct contact with an infected individual or a carrier animal.

Giardiasis may be asymptomatic; however, typical clinical manifestations include diarrhea (acute or chronic), abdominal bloating, abdominal pain, nausea, loss of appetite and body weight, as well as general fatigue (Adam 2021). In pediatric populations, infection may lead to developmental deficiencies and malabsorption syndromes, while in adults it can result in symptoms of irritable bowel syndrome or persistent gastrointestinal disturbances even after the elimination of the parasite (Belkessa et al. 2021; Halliez and Buret 2013).

According to data from the World Health Organization (WHO), giardiasis affects approximately 280 million people globally each year, with the highest incidence observed in developing countries where sanitary conditions are inadequate (Mahdavi et al. 2021). It is estimated that in certain regions of Africa and Asia, up to 20–30% of children may be chronic carriers of *Giardia intestinalis* (Samie et al. 2020). In the United States, giardiasis is among the five most frequently reported parasitic diseases. According to the Centers for Disease Control and Prevention (CDC), over 15,000 cases were reported in 2019; however, the actual number of infections is likely significantly higher due to underdiagnosis of mild or asymptomatic cases (CDC 2025; Coffey et al. 2021).

Giardiasis also represents a significant public health concern in Europe, although epidemiological data from the Central and Eastern European region remain limited. According to 2021 data, 2,169 cases of giardiasis were reported in Germany, 1,042 in Belgium, 591 in Bulgaria, and 1,679 in Spain (Coffey et al. 2021; “Giardiasis - Annual Epidemiological Report for 2021” 2024). In Poland, according to official statistics published on the gov.pl website, 1,771 cases of giardiasis were reported in 2023, representing a significant increase compared to 559 cases in 2021 (Gordat et al. 2024). Furthermore, according to data from the National Institute of Public Health (PZH), 2,145 cases were registered in 2024 (*Choroby zakaźne i zatrucia w Polsce w 2024 roku* 2025). This increase may be attributed to improvements in diagnostic methods, greater public health awareness, and enhanced infectious disease reporting systems. Nevertheless, given that many infections are asymptomatic or present with nonspecific symptoms, and are not always confirmed through laboratory diagnostics, the actual number of infections is most likely significantly higher.

The diagnosis of giardiasis is based on microscopic stool examination, detection of parasite antigens, or polymerase chain reaction (PCR) techniques (Vicente et al. 2024). However, the availability and routine use of advanced diagnostic methods are not yet standard practice in many healthcare facilities, which may contribute to an underestimation of the number of diagnosed cases in routine settings.

Given the increasing clinical significance of giardiasis and the potential underrecognition of infections, there is a pressing need for studies that evaluate infection patterns in clinically tested populations, as well as population-based studies using representative sampling to refine prevalence estimates. Understanding the relationships between health-related behaviors, environmental conditions, and infection rates may facilitate more targeted preventive and diagnostic strategies.

The aim of the present study was to assess the occurrence of *Giardia intestinalis* infection in a clinical, self-selected cohort of individuals undergoing parasitological diagnostics, and to analyze environmental, behavioral and clinical correlates, as well as infection-related symptoms across different age groups in Poland.

## Materials and methods

### Study design and ethical approval

This study was cross-sectional in nature and conducted by the AllerGen laboratory as part of an implementation PhD project funded by the Ministry of Education and Science. The study received approval from the Bioethics Committee (approval number: RNN/27/25/KE, dated January 18, 2025). Participants included patients presenting for intestinal parasite diagnostics, representing a clinically referred, self-selected cohort rather than a population-based sample, who consented to take part in the study and complete an anonymous questionnaire. All participants were informed about the study’s purpose, the voluntary nature of participation, and assured complete anonymity. Questionnaire data were collected between February 2025 and May 2025.

### Description of the survey questionnaire

A custom-designed questionnaire was developed by the research team based on a comprehensive review of the literature and consultations with experts in parasitic diseases. The questionnaire consisted of 23 thematic sections covering demographic information (such as age, place of residence, and occupation), environmental factors (including contact with animals, international travel, and use of public swimming pools), health-related behaviors (such as diet, dietary supplementation, and tobacco use), clinical symptoms potentially associated with *Giardia intestinalis* infection (e.g., diarrhea, changes in body weight, fatigue, concentration disturbances), as well as the history of parasitic infections among family members and in the respondent’s immediate environment. The questionnaire included closed-ended questions with three response options: “YES” or “NO”. For certain symptom-related items, such as types of appetite changes, multiple responses were allowed. Prior to data collection, the questionnaire was pilot-tested in a group of 20 individuals and subsequently administered to patients presenting to the laboratory for parasitological diagnostics.

### Participant eligibility criteria

Inclusion criteria for the study were: age ≥ 6 years, completion of laboratory testing for intestinal parasites at the AllerGen laboratory, and a correctly completed questionnaire. Exclusion criteria included incomplete questionnaire data or lack of informed consent to participate in the study. After excluding incomplete forms, responses from 518 participants were included in the final analysis.

### Diagnostic procedures

All study participants underwent parasitological examination of stool samples at a certified parasitological laboratory operated by AllerGen (Piotrkow Trybunalski, Poland). The diagnostic protocol involved microscopic analysis of three consecutive stool samples for the presence of eggs, cysts, and trophozoites of intestinal parasites such as *Giardia intestinalis*, *Blastocystis spp.*, *Endolimax nana*, *Balantidium coli*, and *Ascaris lumbricoides*. Microscopic diagnostics were performed using a direct wet-mount approach. For each examination, a small portion of fresh stool was placed on a glass slide and mixed with a drop of 0.9% saline solution to assess motile trophozoites and helminth eggs. A parallel preparation using Lugol’s iodine was performed to enhance visualization of cyst structures. Each preparation was covered with a coverslip and examined immediately under light microscopy, initially at 100× and subsequently at 400× magnification. No concentration methods (e.g., formalin–ethyl acetate sedimentation, Faust flotation, or Fülleborne technique) were used, as the study aimed to assess organisms detectable by routine direct microscopy, consistent with the laboratory’s standard diagnostic workflow. All samples were evaluated for intestinal protozoa and helminths detectable by this method; however, only taxa confirmed in the examined cohort (*Giardia intestinalis*,* Blastocystis spp.*,* Endolimax nana*,* Balantidium coli*, and *Ascaris lumbricoides*) are reported in the manuscript. Their listing does not imply exclusion of other taxa at the screening stage but reflects the final diagnostic findings. In selected cases, to confirm *G. intestinalis* infection, an additional enzyme-linked immunosorbent assay (ELISA) was performed to detect the presence of *G. intestinalis* antigen in stool, using the Simple Giardia test (Operon S.A., REF 9109020.21.000, Spain).

### Statistical data analysis

The questionnaire data were coded and subjected to statistical analysis using Microsoft Excel, MedCalc, and SPSS software. Comparisons between infected and non-infected groups were performed using the chi-square test (χ²). For each variable, p-values were calculated, with a significance threshold set at *p* < 0.05. Results were also stratified by age groups: children (6–12 years), adolescents (13–17 years), adults (18–40 years), and older adults (> 40 years). Statistically significant findings were additionally illustrated using heatmaps.

Both univariate and multivariate logistic regression analyses were conducted to assess the strength and independence of associations between individual variables and the presence of *Giardia intestinalis* infection. In the univariate analysis, odds ratios (ORs) with 95% confidence intervals (CIs) were calculated for each independent variable separately. Variables showing statistical significance (*p* < 0.05) were subsequently included in the multivariate analysis to identify independent risk factors. The results of the regression analyses were presented graphically as OR plots, stratified by age group.

It is important to note that the category “other parasites” (*n* = 52; 10.0% of participants) was not included in the statistical analyses due to the small size of this subgroup. Its inclusion could have reduced the power of χ² tests and potentially led to misleading conclusions. Therefore, all comparisons were limited to two categories: non-infected individuals (“no parasite”) and those infected with *Giardia intestinalis*. As participation was driven by clinical referral and voluntary testing, the study cohort does not constitute a random or representative sample of the general population, and analyses should be interpreted accordingly.

## Results

### Participant characteristics

**Fig. 1:**
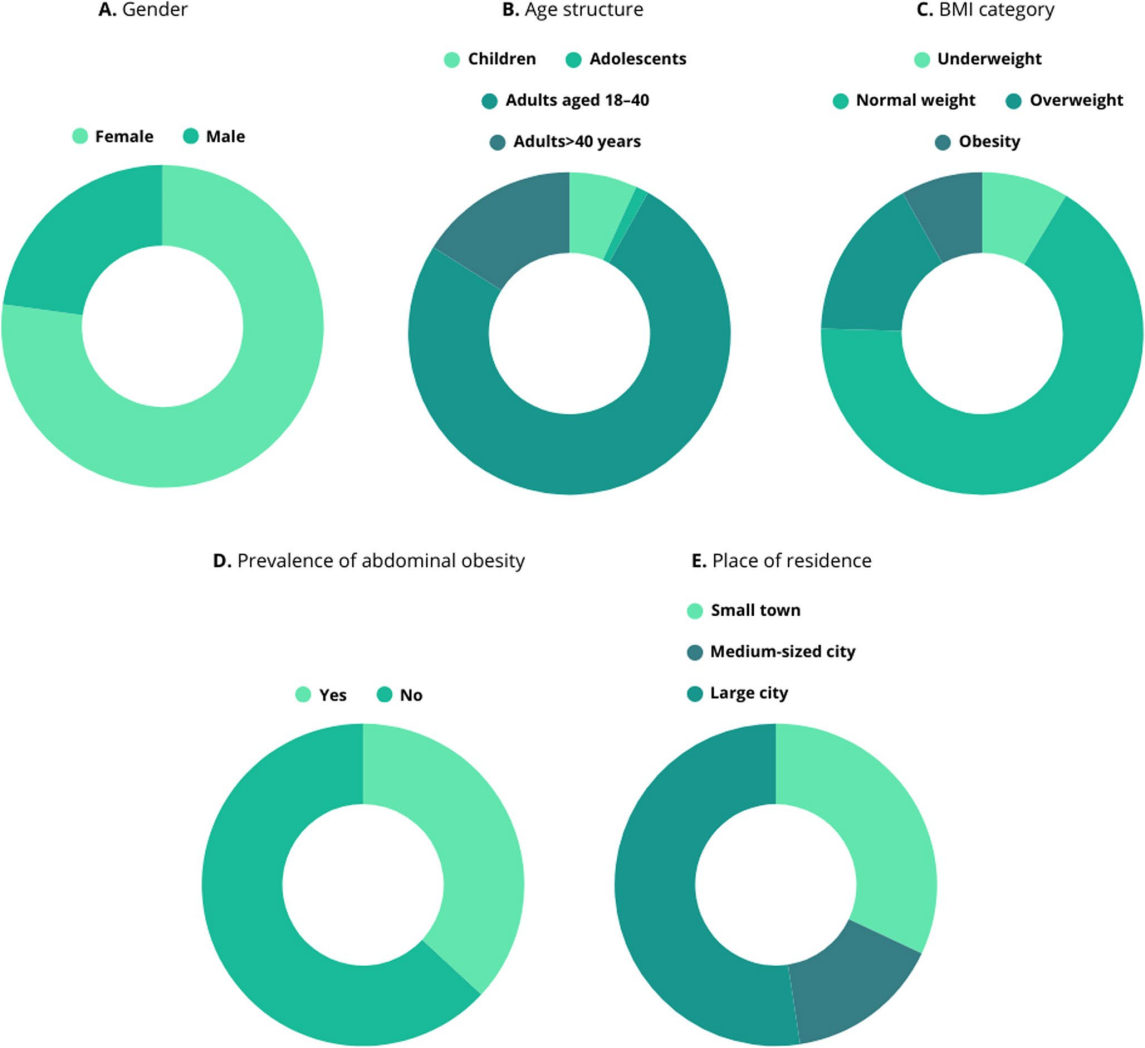
The demographic and anthropometric characteristics of the study population (*n* = 518). **A** - sex distribution; **B** - age structure; **C** - distribution of BMI categories; **D** - prevalence of abdominal obesity; **E** - place of residence of respondents

A total of 518 individuals participated in the study, including 400 women (77.2%) and 118 men (22.8%) (Fig. 1A). The mean age of participants was 31.8 years (SD = 11.46; range: 6–76), with a median of 32.0 years (Table 1). Adults aged 18–40 years comprised the majority of the sample (75.9%, *n* = 394), followed by adults over 40 years (16.0%, *n* = 83), children aged 6–12 years (6.8%, *n* = 35), and adolescents aged 13–17 years (1.3%, *n* = 7) (Fig. 1B). More than half of the participants (52.4%, *n* = 271) resided in large cities, while the remainder lived in small towns (32.0%, *n* = 166) and medium-sized cities (15.6%, *n* = 81) (Fig. 1C).

**Table 1:** Prevalence of *Giardia intestinalis* infection by sex and age group among survey respondents

Variable		No parasites	G. intestinalis	χ²	*p*
Sex	*Female*	64.0% (*n* = 256)	25.5% (*n* = 102)	0.23	0.633
	*Male*	69.5% (*n* = 82)	22.0% (*n* = 26)	0.53	0.468
Group	*Children and adolescents*	78.6% (*n* = 33)	11.9% (*n* = 5)	3.36	0.067
	*Adults (18–40 yrs)*	65.1% (*n* = 256)	24.9% (*n* = 98)	0.002	0.968
	*Adults (> 40 yrs)*	59.0% (*n* = 49)	30.1% (*n* = 25)	1.28	0.257

The presented *p*-values correspond to chi-square tests comparing the distribution of infected vs. non-infected individuals within the analyzed groups

Anthropometric variables (body mass index, percentile scores in minors, waist and hip circumference, and waist-to-hip ratio) were recorded for descriptive purposes and are presented in Supplementary Tables S1–S3.

### Detectable intestinal parasites in the clinical cohort

**Fig. 2:**
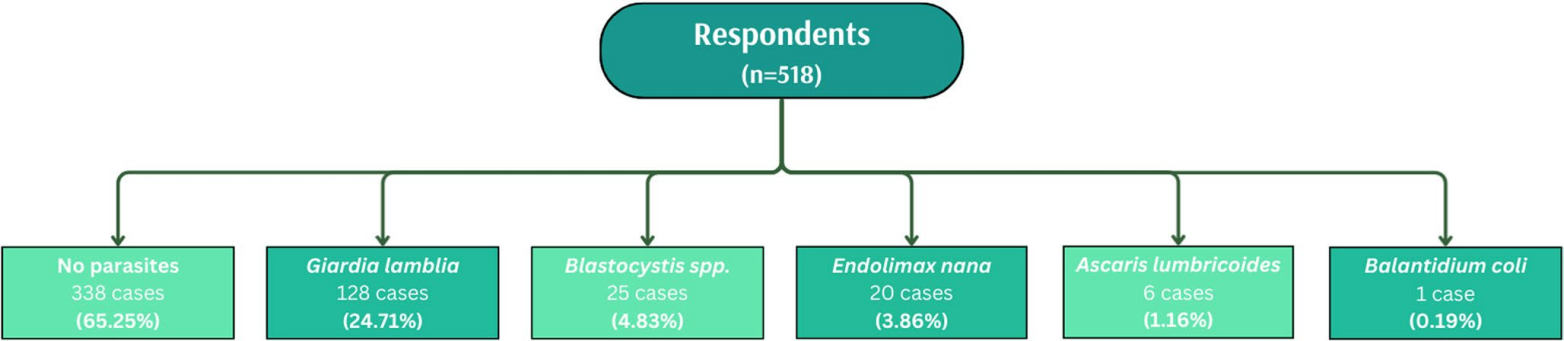
Distribution of detected intestinal parasites in the study population (*n* = 518) The diagram presents the numerical and percentage distribution of individual species of intestinal parasites identified in stool samples obtained from participants of the survey-based study

Stool samples from 518 participants in this clinically referred, self-selected cohort were examined for the presence of intestinal parasites. Parasitic organisms were detected in 180 individuals (34.75%), while no parasites were identified in 338 participants (65.25%) (Fig. 2). The most frequently detected species was *Giardia intestinalis*, identified in 128 individuals (24.71%).

Other parasites were observed less frequently. *Blastocystis spp.* was detected in 25 participants (4.83%), *Endolimax nana* in 20 participants (3.86%), *Ascaris lumbricoides* in 6 participants (1.16%), and *Balantidium coli* in 1 participant (0.19%) (Fig. 2).

Subsequent statistical analyses focused on *G. intestinalis*, which was the most frequently detected taxon in this clinically tested cohort.

### Age-specific distribution of giardia intestinalis infections in the clinical cohort

Infections were detected in 25.5% of women (*n* = 102) and 22.0% of men (*n* = 26) within the clinical cohort, with no statistically significant differences between sexes (χ²=0.23, *p* = 0.633; χ²=0.53, *p* = 0.468).

The prevalence of *G. intestinalis* infection across age groups was 11.9% among children and adolescents (*n* = 5), 24.9% among adults aged 18–40 years (*n* = 98), and 30.1% among adults over 40 years (*n* = 25). These differences were not statistically significant (χ²=3.36, *p* = 0.067; χ²=0.002, *p* = 0.968; χ²=1.28, *p* = 0.257, respectively) (Table 1).

### Risk factors for giardia intestinalis infection

**Fig. 3:**
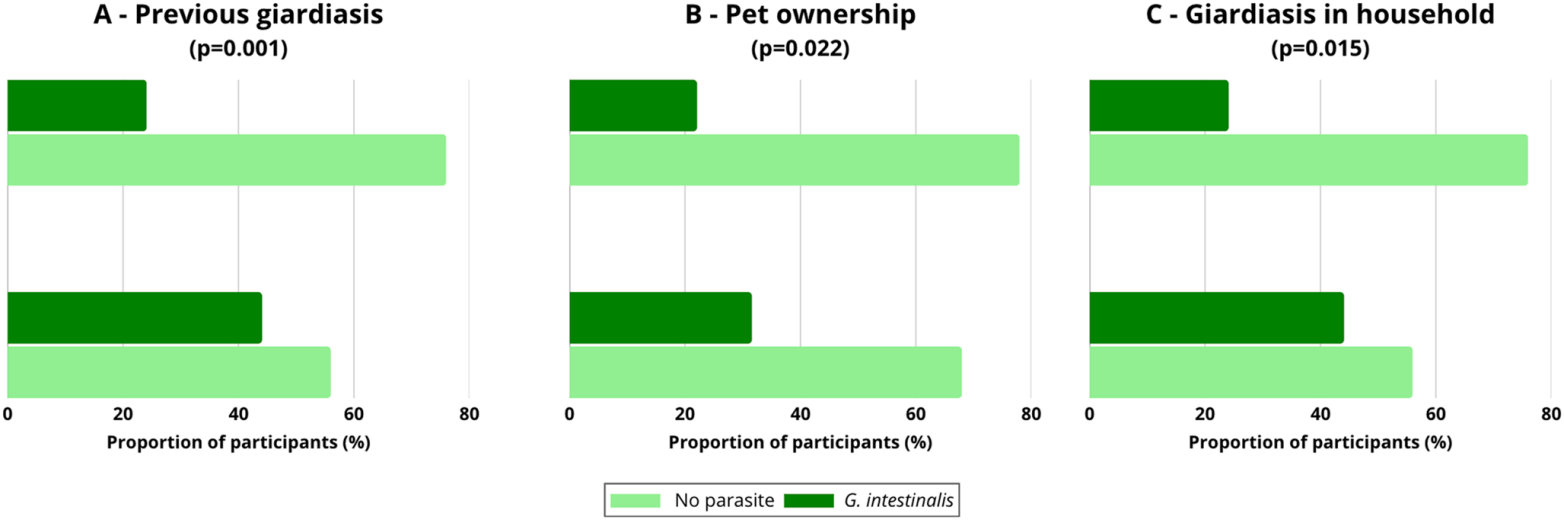
Frequency of *Giardia intestinalis* infections according to statistically significant factors: (**A**) previous giardiasis, (**B**) pet ownership, (**C)** occurrence of giardiasis in the household. The proportion of participants infected with *G. intestinalis* is presented in comparison to those without parasites. Chi-square test; p-values are provided in the panel titles

Associations between *G. intestinalis* infection and environmental, clinical, and lifestyle variables were assessed for the entire study population (Fig. 3). Three variables showed statistically significant associations (*p* < 0.05).

Participants with a previous history of giardiasis had a higher infection frequency (44.1%; *n* = 30) compared to those without prior infection (24.1%; *n* = 86) (χ²=11.55; *p* = 0.001) (Fig. 3A). Pet ownership was also associated with a higher infection frequency (31.6%; *n* = 81) compared to participants without pets (22.1%; *n* = 46) (χ²=5.24; *p* = 0.022) (Fig. 3B). Additionally, infection was more frequent among individuals reporting giardiasis cases in their household (41.7%; *n* = 20) compared to those without household exposure (25.0%; *n* = 83) (χ²=5.90; *p* = 0.015) (Fig. 3C).

Several variables showed non-significant trends, including residence in small towns (*p* = 0.063), dietary changes (*p* = 0.070), previous surgical procedures (*p* = 0.073), and regular medical check-ups (*p* = 0.069). No statistically significant differences were observed for sex, age group, smoking, dietary habits (including consumption of raw or unwashed foods or untreated water), previous hospitalizations, or chronic diseases (*p* > 0.05).

**Fig. 4:**
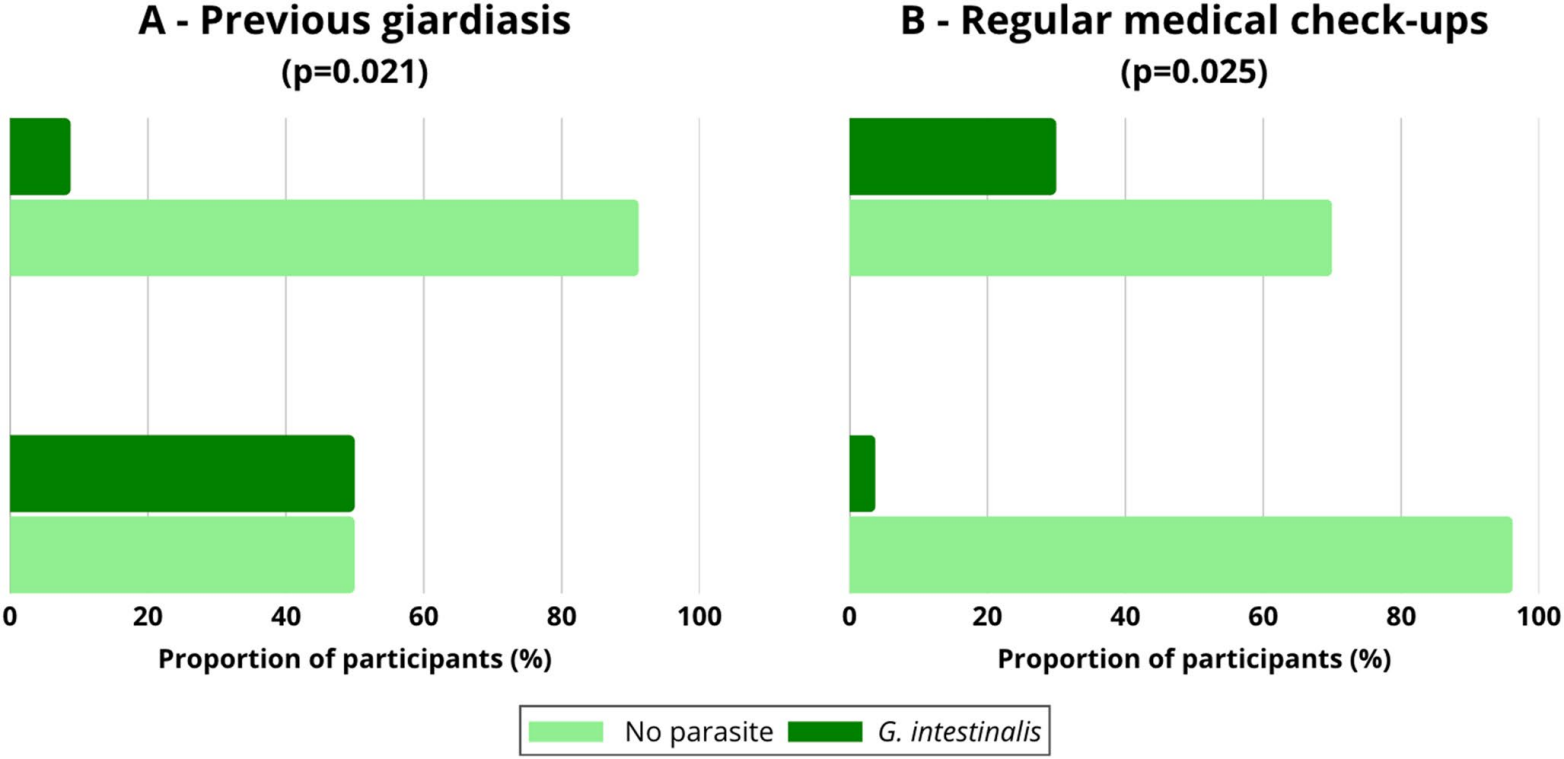
Frequency of *Giardia intestinalis* infections in the subgroup of children and adolescents according to statistically significant factors: (**A**) previous giardiasis and (**B**) regular medical check-ups. The proportion of participants infected with *G. intestinalis* is presented in comparison to those without parasites. Chi-square test; p-values are provided in the panel titles. The small sample size limits the generalizability of the results

In the subgroup of children and adolescents (*n* = 38), two statistically significant associations were observed (Fig. 4). Infection occurred more frequently in individuals with previous giardiasis (50.0%; 2/4) compared to those without such history (8.8%; 3/34) (χ²=5.31; *p* = 0.021) (Fig. 4A). Infection was also more frequent in participants not undergoing regular medical check-ups (30.0%; 3/10) compared to those who reported regular examinations (3.8%; 1/26) (χ²=5.00; *p* = 0.025) (Fig. 4B).

**Fig. 5:**
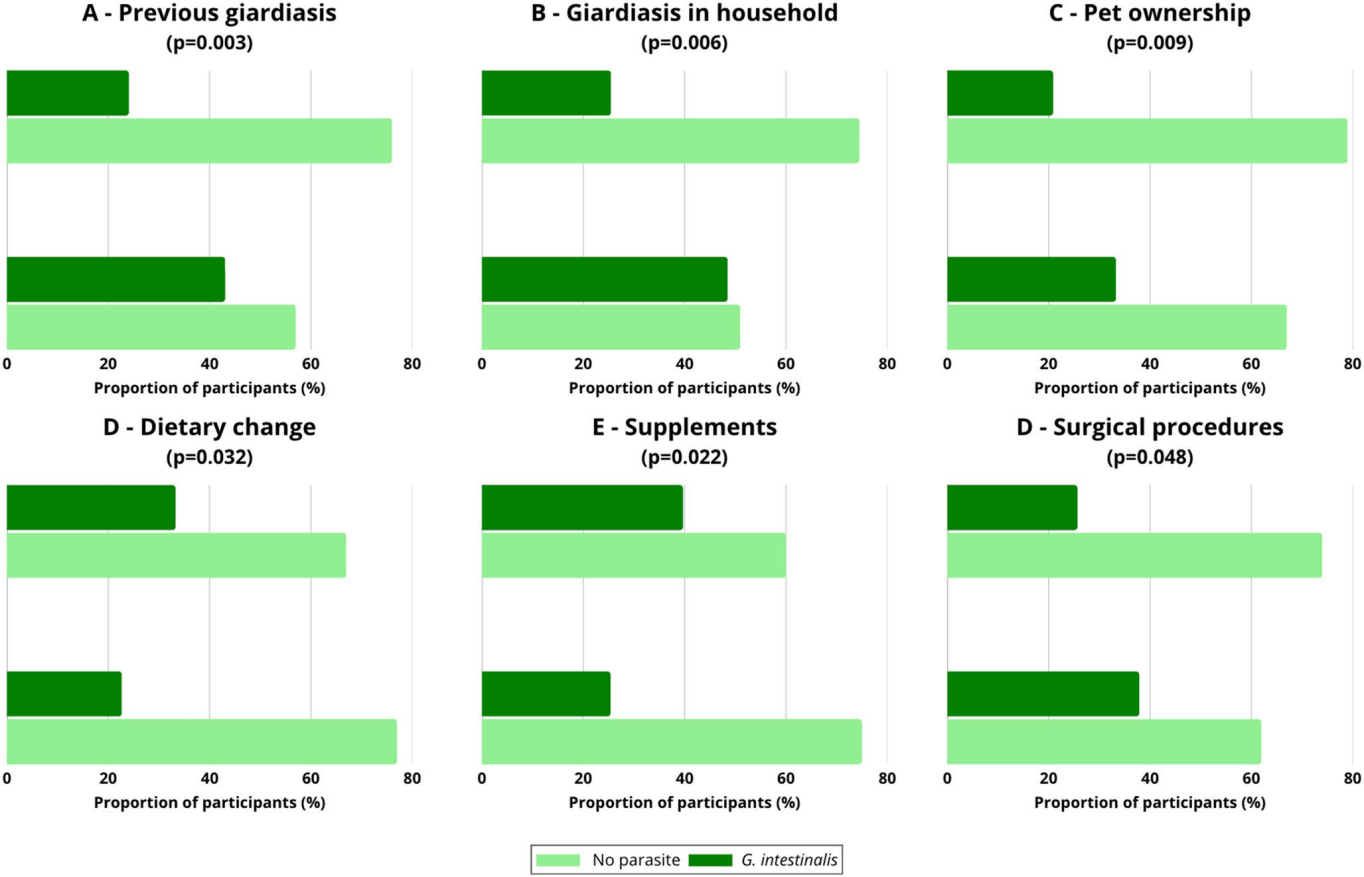
Frequency of *Giardia intestinalis* infections in adults (18–40 years) according to statistically significant factors: (**A**) previous giardiasis, (**B**) giardiasis in the household, F (**C**) pet ownership, (**D**) dietary change or modification of eating habits, (**E**) supplement intake, and (**F**) history of surgical procedures. The proportion of participants infected with *G. intestinalis* is presented in comparison to those without parasites. Chi-square test; p-values are provided in the panel titles

In adults aged 18–40 years, significant associations were observed for previous giardiasis (43.1% vs. 24.0%; χ²=8.65; *p* = 0.003), household cases (48.5% vs. 25.5%; χ²=7.67; *p* = 0.006), and pet ownership (33.3% vs. 20.9%; χ²=6.74; *p* = 0.009) (Fig. 5A-C). Additional associations were observed for dietary changes (22.7% vs. 33.3%; χ²=4.59; *p* = 0.032), supplement intake (25.4% vs. 39.7%; χ²=5.22; *p* = 0.022), and history of surgical procedures (37.9% vs. 25.7%; χ²=3.92; *p* = 0.048) (Fig. 5D-F).

**Fig. 6:**
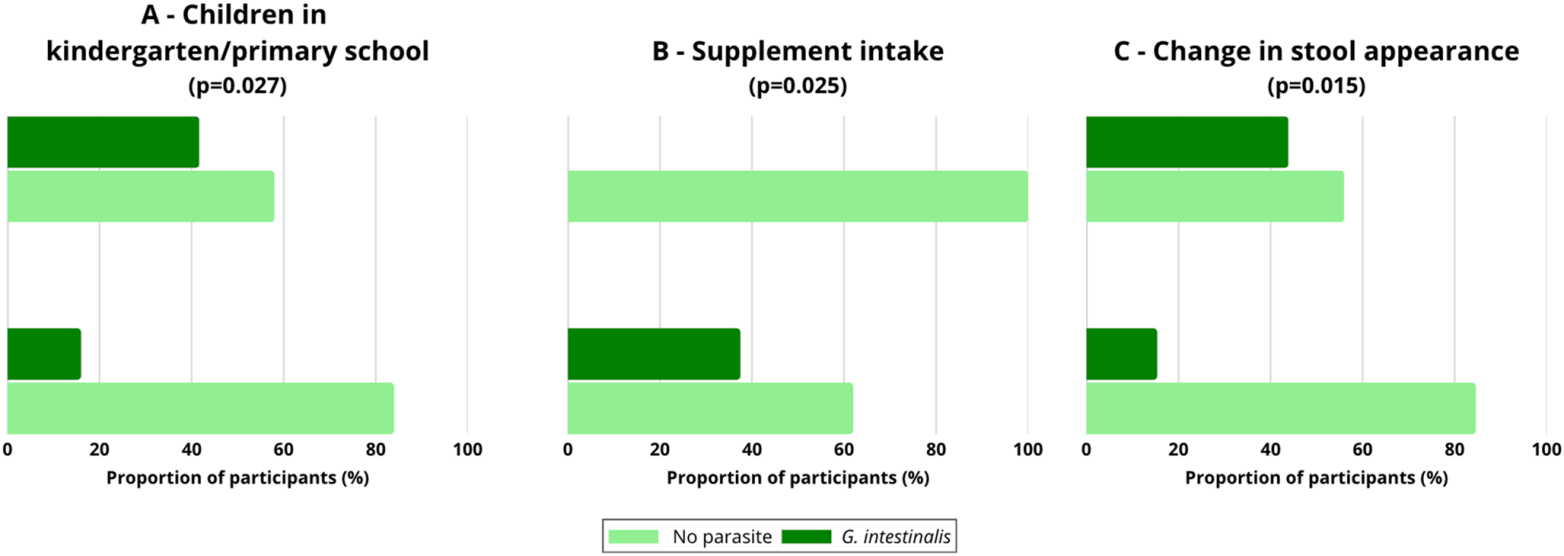
Frequency of *Giardia intestinalis* infections in individuals > 40 years according to statistically significant factors: (**A**) children attending kindergarten or primary school, (**B**) supplement intake, and (**C**) change in stool appearance. The proportion of participants infected with *G. intestinalis* is presented in comparison to those without parasites Chi-square test; p-values are provided in the panel titles

In adults over 40 years, significant associations were found for having children in preschool or primary school (16.0% vs. 41.7%; χ²=4.91; *p* = 0.027), supplement intake (37.5% vs. 0%; χ²=5.03; *p* = 0.025), and reporting changes in stool characteristics (15.4% vs. 43.9%; χ²=5.87; *p* = 0.015) (Fig. 6A-C).

### Clinical symptoms associated with giardia intestinalis infection

**Fig. 7:**
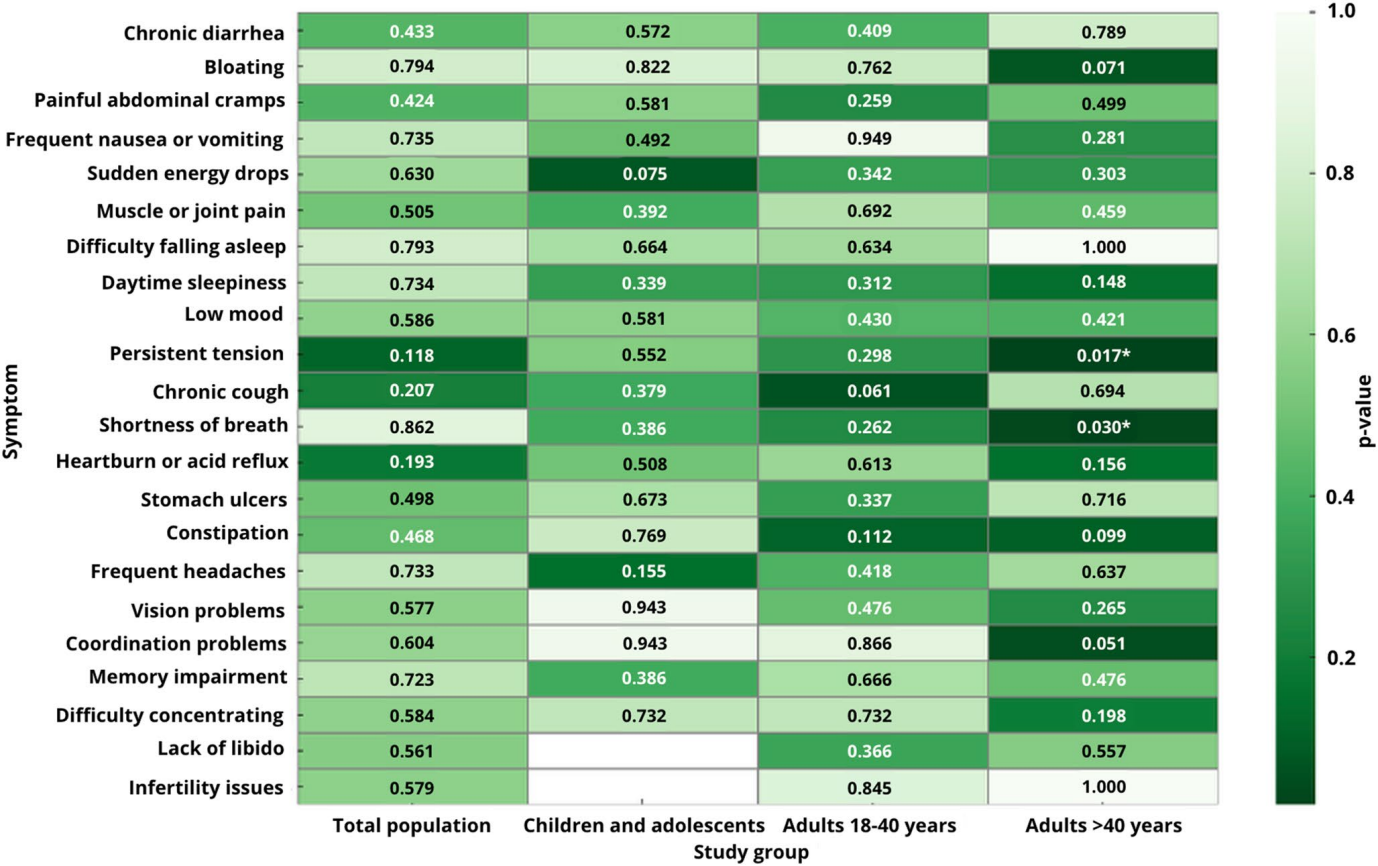
Composite heatmap of *p*-values for associations between clinical symptoms and *Giardia intestinalis* infection in the total study population and in subgroups: children and adolescents, adults aged 18–40 years, and adults over 40 years of age

Clinical symptoms were evaluated in relation to *G. intestinalis* infection across the entire study population (Fig. 7). No statistically significant associations were observed for any of the assessed symptoms (all *p* > 0.05).

In children and adolescents (*n* = 38), none of the analyzed symptoms showed statistically significant associations with infection (*p* > 0.05) (Fig. 7).

In adults aged 18–40 years, no statistically significant associations were found between infection and reported symptoms (*p* > 0.05), although percentage differences were observed for selected variables, including chronic cough (*p* = 0.061) and constipation (*p* = 0.112) (Fig. 7).

In adults over 40 years of age, three symptoms demonstrated statistically significant associations with infection: persistent tension (46.2% vs. 19.0%; χ²=5.68; *p* = 0.017), dyspnea (38.5% vs. 0.0%; χ²=4.73; *p* = 0.030), and constipation (37.7% vs. 16.7%; χ²=5.68; *p* = 0.017) (Fig. 7).

### Logistic regression analysis results – total study population and age subgroups

**Fig. 8:**
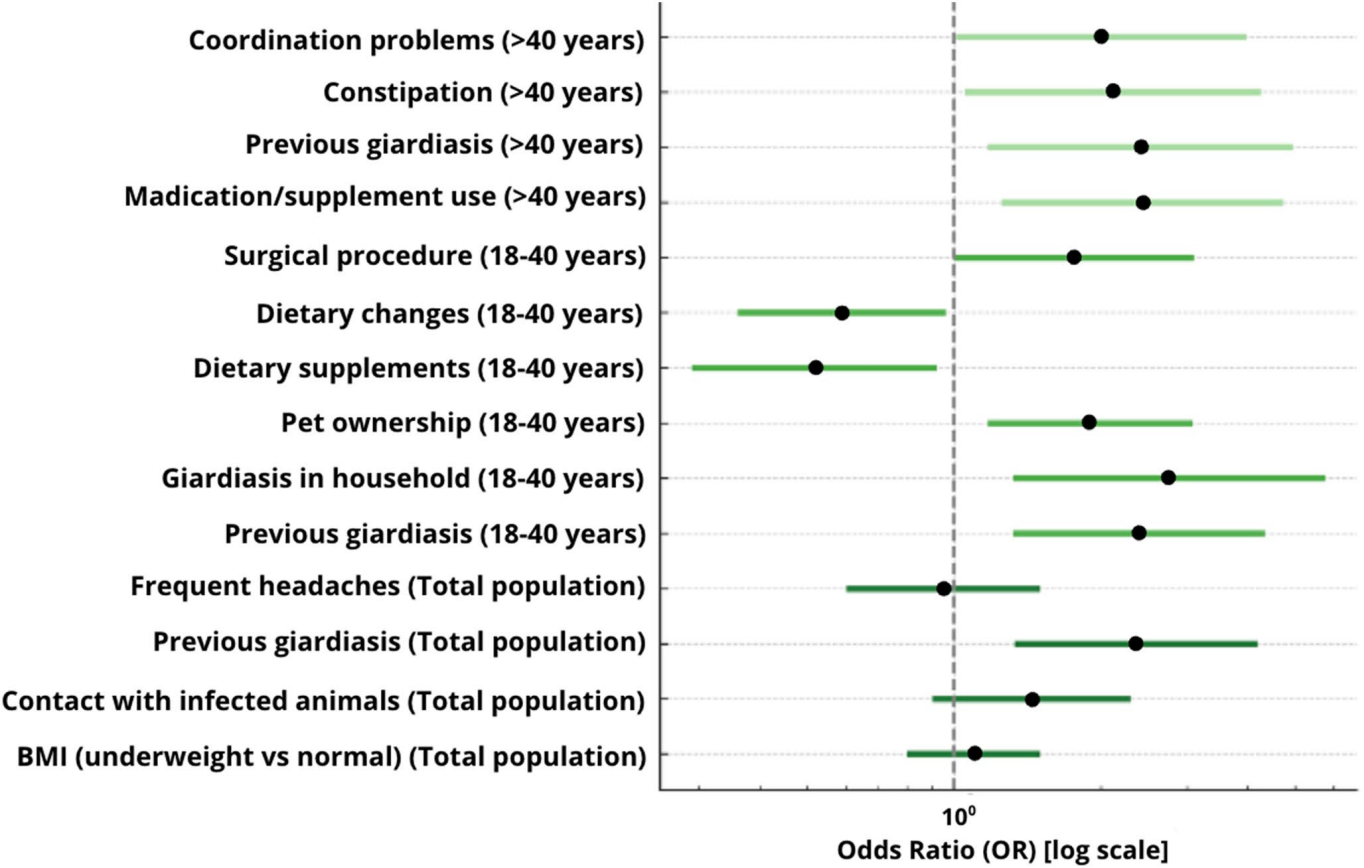
Forest plot presenting the results of univariate logistic regression analysis for *Giardia intestinalis* infection in the total study population and in age-stratified subgroups (18–40 years and > 40 years). Odds ratios (OR) are shown with 95% confidence intervals, with the vertical dashed line indicating the reference value (OR = 1). The horizontal axis is displayed on a logarithmic scale. Only variables that reached statistical significance (*p* < 0.05) in at least one of the analyzed groups are presented. Dark green bars correspond to the total study population, medium green to adults aged 18–40 years, and light green to adults > 40 years

Univariate logistic regression analyses were performed for the total study population and separately for adults aged 18–40 years and individuals over 40 years (Fig. 8).

In the total study population, statistically significant associations with *G. intestinalis* infection were observed for previous giardiasis (OR = 2.36; 95% CI: 1.33–4.18; *p* = 0.003), household cases of giardiasis (OR = 2.11; 95% CI: 1.24–3.59; *p* = 0.006), and pet ownership (OR = 1.81; 95% CI: 1.16–2.82; *p* = 0.009). Lower odds of infection were observed among participants reporting supplement intake (OR = 0.55; 95% CI: 0.33–0.90; *p* = 0.022) and dietary modifications (OR = 0.59; 95% CI: 0.36–0.97; *p* = 0.032). Higher odds were observed for individuals with a history of surgical procedures (OR = 1.76; 95% CI: 1.01–3.05; *p* = 0.048). Waist circumference was positively associated with infection risk as a continuous variable (OR = 1.03; 95% CI: 1.00–1.05; *p* = 0.030).

In adults aged 18–40 years, significant associations were observed for previous giardiasis (OR = 2.39; 95% CI: 1.32–4.33; *p* = 0.004), household cases (OR = 2.75; 95% CI: 1.32–5.76; *p* = 0.007), pet ownership (OR = 1.89; 95% CI: 1.17–3.08; *p* = 0.010), supplement use (OR = 0.52; 95% CI: 0.29–0.92; *p* = 0.024), and dietary changes (OR = 0.59; 95% CI: 0.36–0.96; *p* = 0.033). A borderline association was observed for prior surgical procedures (OR = 1.76; 95% CI: 1.00–3.10; *p* = 0.051).

In participants over 40 years of age, significant associations were observed for regular use of medications or supplements (OR = 2.43; 95% CI: 1.25–4.72; *p* = 0.009), previous giardiasis or other parasitic infections (OR = 2.41; 95% CI: 1.17–4.95; *p* = 0.017), constipation (OR = 2.11; 95% CI: 1.05–4.25; *p* = 0.036), and reported problems with motor coordination (OR = 2.00; 95% CI: 1.01–3.97; *p* = 0.048).

**Fig. 9:**
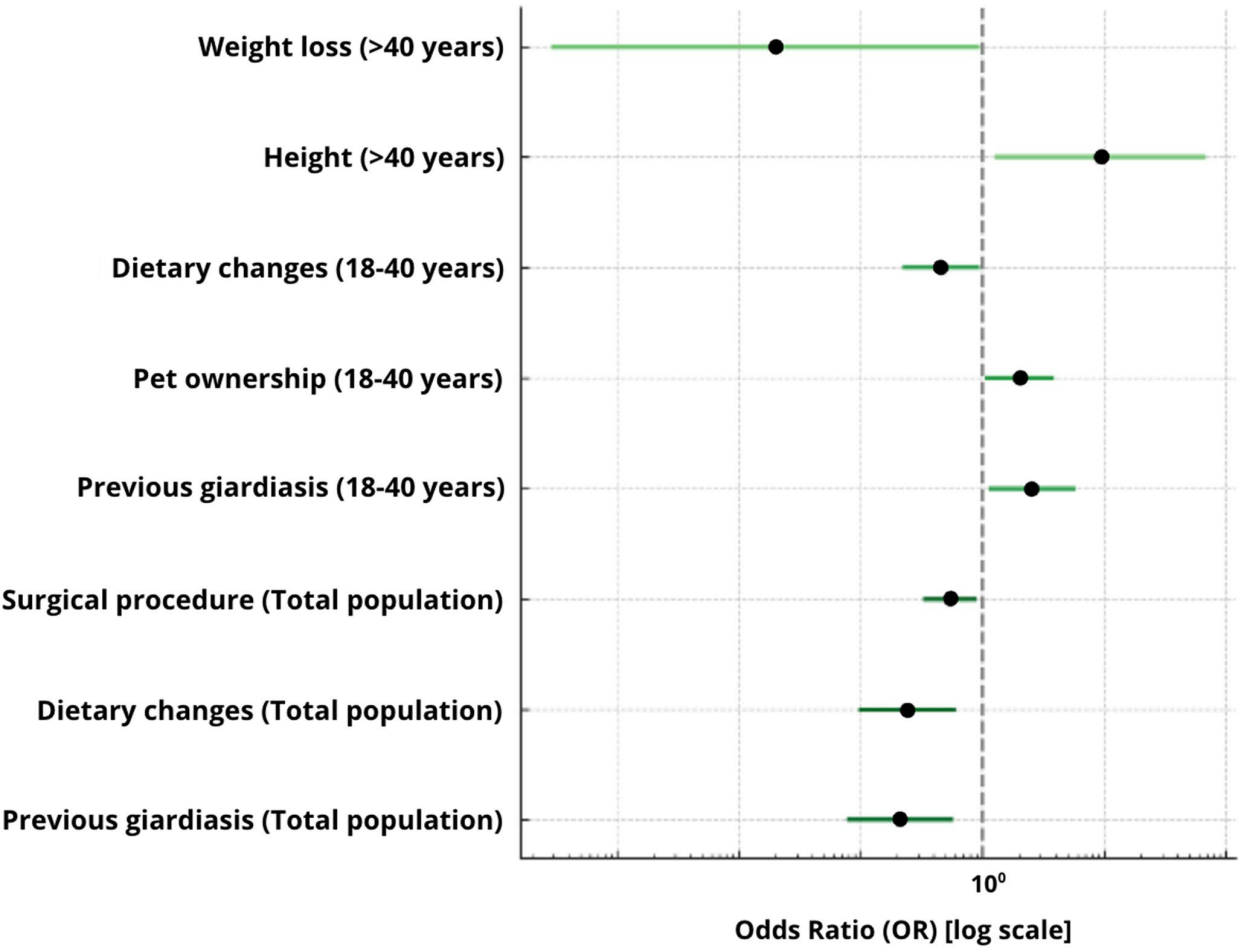
Forest plot presenting the results of multivariate logistic regression analysis for *Giardia intestinalis* infection in the total study population and in age-stratified subgroups (18–40 years and > 40 years). Odds ratios (OR) are shown with 95% confidence intervals, with the vertical dashed line indicating the reference value (OR = 1). The horizontal axis is displayed on a logarithmic scale. Only variables that reached statistical significance (*p* < 0.05) in at least one of the models are presented. Dark green bars correspond to the total study population, medium green to adults aged 18–40 years, and light green to adults > 40 years

Multivariate logistic regression analysis identified independent associations in the total population (Fig. 9). Lower odds of infection were found for supplement intake (OR = 0.21; 95% CI: 0.08–0.55; *p* = 0.001), bloating (OR = 0.24; 95% CI: 0.10–0.58; *p* = 0.001), and having children attending kindergarten or primary school (OR = 0.38; 95% CI: 0.15–0.95; *p* = 0.038). Higher odds were observed for constipation (OR = 2.22; 95% CI: 1.06–4.63; *p* = 0.034).

In adults aged 18–40 years, independent associations were retained for previous giardiasis (OR = 2.57; 95% CI: 1.17–5.64; *p* = 0.018), pet ownership (OR = 2.00; 95% CI: 1.09–3.69; *p* = 0.026), and dietary changes (OR = 0.46; 95% CI: 0.23–0.91; *p* = 0.026).

In participants over 40 years of age, independent associations were observed for body height (OR = 9.29 per 1 SD increase; 95% CI: 1.33–64.98; *p* = 0.025) and recent weight loss (OR = 0.02; 95% CI: 0.0003–0.91; *p* = 0.045).

## Discussion

In this clinically referred, self-selected cohort, *Giardia intestinalis* infection was detected in 24.7% of participants. While this proportion is numerically higher than figures reported in national surveillance systems, the non-representative sampling design precludes inferences about prevalence in the general population. According to official gov.pl statistics, 1,771 cases were reported in 2023, compared with 559 in 2021, whereas the National Institute of Public Health reported 2,145 cases in 2024 (Gordat et al. 2024; *Choroby zakaźne i zatrucia w Polsce w 2024 roku* 2025). Differences between routine reporting and proportions observed in clinically tested groups likely reflect diagnostic referral patterns, underrecognition of asymptomatic cases, and limited access to testing rather than population-level prevalence disparities.

Similar patterns have been reported in Norway, the United States, and Canada, where screening of symptomatic or high-risk individuals yielded higher detection rates than routine surveillance (Coffey et al. 2021; Hanevik et al. 2009; Painter et al. 2012).

These discrepancies may result from asymptomatic or nonspecific presentation of giardiasis, limited routine testing, and insufficient clinical awareness (Vicente et al. 2024). The predominance of *G. intestinalis* among detected parasites in this cohort further supports its clinical relevance and justified focusing subsequent analyses on this species.

Adults aged 18–40 and > 40 years showed higher frequencies of infection compared with children and adolescents within the cohort, although these differences were not statistically significant. This pattern may reflect the age structure of individuals seeking diagnostic testing, differential exposure, or immunity-related factors across age groups (Doherty et al. n.d.; Muhsen and Levine 2012). The higher proportion of adults in the sample may also have influenced this pattern. The absence of symptom associations in younger adults is consistent with reports indicating that giardiasis in this age group may be asymptomatic or nonspecific, whereas older adults more frequently reported constipation, dyspnea, and tension.

Across regression models, previous giardiasis, household exposure, and pet ownership consistently increased infection odds, particularly in younger adults. These findings are aligned with literature describing intrafamilial clusters and animal contact as major transmission pathways in high-income regions. Reinfection among previously infected individuals may indicate repeated exposure, persistent environmental contamination, or host susceptibility and highlights the need for follow-up testing rather than single-episode treatment.

Health-related behaviors showed heterogeneous associations. Dietary modifications remained independently associated with lower odds of infection in younger adults, while supplements and bloating were associated with reduced odds in the overall model. The role of supplement intake requires age-stratified interpretation. In the total cohort, supplement use was associated with lower odds of infection in both univariate and multivariate models, which may reflect health-conscious behavior, dietary modification, or other characteristics correlated with lower exposure risk rather than a direct biological effect. In contrast, among individuals > 40 years, supplement use was associated with a higher proportion of infections. This pattern may reflect the underlying health profile of this subgroup, as supplement intake in older adults is frequently motivated by chronic gastrointestinal and metabolic conditions that may themselves lead to more frequent diagnostic testing, thereby increasing the likelihood of infection detection. Additionally, supplement use may co-occur with medication regimens or altered gut physiology, which could influence susceptibility to infection or symptom-driven testing. Therefore, supplement intake should not be interpreted as biologically protective or harmful per se, but rather as a proxy for distinct behavioral and clinical characteristics that vary by age group. These associations reflect patterns within the tested cohort and should not be interpreted causally without longitudinal data.

The inverse association between bloating and *Giardia intestinalis* infection observed in the multivariate model (OR = 0.24; *p* = 0.001) was unexpected, as bloating is typically considered a hallmark symptom of giardiasis. Given that data coding was verified and symptom variables were entered correctly, this result likely reflects factors related to clinical referral patterns within this self-selected cohort rather than a true protective effect. Individuals reporting bloating may be more likely to seek medical testing for gastrointestinal complaints unrelated to giardiasis, resulting in overrepresentation of non-infected but symptomatic individuals in the control group. In addition, bloating frequently co-occurs with conditions such as small intestinal dysbiosis, food intolerances, or functional gastrointestinal disorders, which are overdiagnosed in settings where patients self-refer for testing and may present similarly to parasitic infections. These factors may attenuate or reverse the association between bloating and infection when other exposure-related predictors are included in multivariate models. Another plausible explanation is multicollinearity with other symptom variables, such as constipation, which showed a positive association with infection in the same model. Although the present findings do not support using bloating as a discriminatory clinical predictor of giardiasis in clinically referred populations, they highlight heterogeneity of symptom presentation and suggest that symptoms alone may not reliably distinguish infected from non-infected individuals. Further research using population-based sampling and standardized clinical assessment is needed to clarify whether this pattern reflects methodological factors or symptom-specific host responses.

Regression models suggested that correlates of infection differed by age. In younger adults, exposure-related and lifestyle factors predominated, whereas in older adults associations emerged with anthropometric parameters, medication or supplement use, constipation, and motor coordination difficulties. Anthropometric associations should be interpreted cautiously due to limited sample size and lack of population-level representativeness.

The effect of prior giardiasis varied by age group and analytical model. Lower adjusted odds of infection in the total population may indicate acquired or partial immunity or behavioral modification following treatment. In contrast, in younger adults, previous giardiasis increased the odds of reinfection, consistent with repeated exposure or persistent susceptibility. Similar divergent effects have been reported elsewhere (Halliez and Buret 2013; Cacciò and Ryan 2008), suggesting that outcomes depend on host, parasite, and environmental interactions. Longitudinal studies are needed to clarify whether previous infection confers protection, signals reinfection, or marks continued exposure.

Experimental and clinical research indicates that giardiasis may disrupt immune pathways, metabolism, and gut microbiota composition, potentially influencing neurogastroenterological consequences (Halliez and Buret 2013; Konishi et al. 2024). Persistent post-infectious symptoms, including IBS and chronic fatigue, have been documented in long-term follow-up cohorts (Hanevik et al. 2014). In children, associations with growth impairment and micronutrient deficiencies have been reported independent of inflammation (Giallourou et al. 2023; Belkessa et al. 2021). These findings underscore the systemic nature of giardiasis and highlight the importance of post-treatment follow-up in high-risk groups.

This study was conducted among individuals seeking parasitological testing and is not representative of the general population. Adults and urban residents were overrepresented, limiting generalizability to rural and pediatric populations. Cross-sectional design precludes inference of causality, and exposure data were self-reported and subject to recall bias. Subgroup analyses, particularly in children and adults over 40 years, included relatively small samples, reducing statistical power and stability of estimates.

Microscopic diagnostics relied exclusively on direct wet-mount methods without concentration techniques, which may underestimate low-intensity infections and limit comparability with studies using standardized surveillance protocols recommended by WHO/CDC.

This study demonstrates a high proportion of *G. intestinalis* infection among individuals undergoing parasitological diagnostics in Poland and identifies multiple associated factors within this clinical cohort. These findings underscore the need for improved diagnostic vigilance in symptomatic patients and targeted preventive strategies. Population-based studies using standardized diagnostic protocols are required to determine prevalence and clarify causal pathways.

## Supplementary Information

Below is the link to the electronic supplementary material.

Supplementary Material 1 (PDF 157 KB)

## Data Availability

The datasets generated and/or analyzed during the current study are not publicly available due to participant confidentiality but are available from the corresponding author on reasonable request.

## Declarations

### Clinical trial registration

Clinical trial number: not applicable.
