# Supplementary material for: Analysis of environmental risk factors, prevalence, and associated symptoms of Giardia intestinalis infection across age groups in Poland: a survey-based study
Source: Parasitol Res. 2026 Feb 18;125(1):28. doi: 10.1007/s00436-026-08631-3 (PMC12920409; doi:10.1007/s00436-026-08631-3)
Supplement: Supplementary file 1 — Supplementary Material 1 (PDF 157 KB) [file 436_2026_8631_MOESM1_ESM.pdf]

Supplementary Information
Article title: *Analysis of Environmental Risk Factors, Prevalence, and Associated Symptoms of Giardia intestinalis Infection Across Age Groups in Poland: A Survey-Based Study*
Journal: Parasitology Research
Authors: Sylwia Klimczak[1,2], Kacper Packi[1,2], Alicja Rudek[2], Marcin Kurowski[3], Agnieszka Śliwińska[1,*]
Corresponding author: agnieszka.sliwinska@umed.lodz.pl
Affiliations:
[1] Department of Nucleic Acid Biochemistry, Medical University of Lodz, 92-213 Lodz, Poland.
[2] AllerGen Center of Personalized Medicine, 97-300 Piotrkow Trybunalski, Poland.
[3] Department of Immunology and Allergy, Medical University of Lodz, 92-213 Lodz, Poland.

Online Resource 1 – Table S1. BMI classification in adults

Table S1. Measures of central tendency and dispersion — age of study participants, n = 518

Variable	Mean	SD	Q_1	Me	Q_3	Mo	Min.	Max.
**Age [years]**	31.8	11.46	27.0	32.0	37.0	32.0	6.0	76.0

Mean – arithmetic mean, SD – standard deviation, Me – median, Mo – mode, Min. – minimum value, Max. – maximum value

Online Resource 2 – Table S2. Anthropometric characteristics in adults

Table S2. Anthropometric profile of participants.

Variable	Mean	SD	Q_1	Me	Q_3	Mo	Min.	Max.
**Height [cm]** n=513	165.1	16.48	162.0	168.0	173.0	170.0	110.0	200.0
**Weight [kg]** n=512	63.0	18.18	54.0	62.0	74.0	60.0	12.0	131.0
**BMI*** n=473	23.2	4.32	20.3	22.2	25.0	24.2	15.4	47.3
**Centiles**** n=39	33.4	30.72	8.1	17.1	48.7	42.0	0.3	99.3

Mean – arithmetic mean, SD – standard deviation, Me – median, Mo – mode, Min. – minimum value, Max. – maximum value

*BMI calculated for participants aged 18+; ** Centile values for participants under 18

Online Resource 3 – Table S3. Percentile scores in minors

Table S3. Waist and hip circumference, WHR index among participants aged over 18 years.

Variable	Mean	SD	Q_1	Me	Q_3	Mo	Min.	Max.
**Waist circ. [cm]** n=364	77.6	13.23	69.0	75.5	86.0	70.0	49.0	140.0
**Hip circ. [cm]** n=363	94.0	12.07	88.0	94.0	101.0	88.0	52.0	132.0
**WHR index** n=360	0.8	0.12	0.8	0.8	0.9	0.8	0.6	1.8

Mean – arithmetic mean, SD – standard deviation, Me – median, Mo – mode, Min. – minimum value, Max. – maximum value
